# Prognostic significance of *FSCN* family in multiple myeloma

**DOI:** 10.7150/jca.53675

**Published:** 2021-01-30

**Authors:** Cong Deng, Chaozeng Si, Xu Ye, Qiang Zhou, Tiansheng Zeng, Zeyong Huang, Wenhui Huang, Pei Zhu, Qingfu Zhong, Zhihua Wu, Huoyan Zhu, Qing Lin, Wenjuan Zhang, Lin Fu, Yongjiang Zheng, Tingting Qian

**Affiliations:** 1 Department of Clinical laboratory, The Second Affiliated Hospital, Guangzhou Medical University, 510260 Guangzhou, China.; 2 Department of Information Center, China-Japan Friendship Hospital, 100029 Beijing, China.; 3 Department of Hematology, The Second Affiliated Hospital, Guangzhou Medical University, 510260 Guangzhou, China.; 4 Translational Medicine Center, State Key Laboratory of Respiratory Disease, The Second Affiliated Hospital of Guangzhou Medical University, 510260 Guangzhou, China.; 5 Guangdong Provincial Education Department Key Laboratory of Nano-Immunoregulation Tumor Microenvironment, The Second Affiliated Hospital of Guangzhou Medical University, 510260 Guangzhou, China.; 6 Translational Medicine Center, Huaihe Hospital of Henan University, 475000 Kaifeng, China.; 7 Department of Hematology, Huaihe Hospital of Henan University, 475000 Kaifeng, China.; 8 Department of Hematology, Institute of Hematology, The Third Affiliated Hospital of Sun Yat-Sen University, 510630 Guangzhou, China.

**Keywords:** *FSCN*, multiple myeloma, biomarker, prognosis.

## Abstract

Multiple myeloma (MM) is a hematologic tumor with monoclonal proliferation of malignant plasma cells in the bone marrow. Fascin (FSCN) is an actin-binding protein that plays a crucial role in cell migration and invasion, contributing to tumor metastasis. There are three members (*FSCN1-3*) in *FSCN* family*.* However, the prognostic role of *FSCN* family in MM remains unclear. In this study, we used four independent Gene Expression Omnibus (GEO) datasets to explore the relationships between *FSCN1-3* expression profiles and patient survival in MM. We found that *FSCN1* was dramatically down-regulated in MM compared to normal donors (*p* < 0.001) and monoclonal gammopathy of undetermined significance (MGUS) (*p* = 0.032). Patients with high expression of *FSCN1* and *FSCN2* had significantly longer OS (*p* = 0.023 and 0.028, respectively). Univariate and multivariate analysis showed that *FSCN1* (*p* = 0.003, 0.002) and* FSCN2* (*p* = 0.018, 0.013) were independent favorable prognostic factors for OS in MM. Moreover, the combination of high expression of *FSCN1* and* FSCN2* could effectively predict both longer EFS (*p* = 0.046) and OS (*p* = 0.015). Our study suggested that *FSCN1* and* FSCN2* can be used as favorable biomarkers for predicting clinical outcomes in MM.

## Introduction

Multiple myeloma (MM) is a hematologic malignancy characterized by the expansion of clonal plasma cells in bone marrow and abnormal secretion of immunoglobulins 1. MM can be grouped into asymptomatic or symptomatic based on with or without myeloma-related organ or tissue dysfunction, including hypercalcemia, renal impairment, anemia and bone lesions 1, 2. Monoclonal gammopathy of undetermined significance (MGUS) is considered as an asymptomatic premalignant stage. There are 0.5-1% of MGUS that can evolve into symptomatic MM (intramedullary MM) per year, and may finally progress to extramedullary MM or plasma cell leukemia (PCL) 1, 3. Clinical stage and cytogenetic abnormalities are the most commonly used variables for risk stratification in MM 4. In addition, gene expression profiling has been recognized as an important prognostic factor in recent years 4, 5. Exploring more powerful biomarkers is very meaningful for identifying patients with poor prognosis earlier and providing better therapy strategies, especially for asymptomatic high-risk MM patients 6.

Fascin (FSCN) is a 55-kDa actin-binding protein involved in the formation and stability of microspikes, filopodia and invadopodia, which leads to cell adhesion, motility and migration 7-9. There are three isoforms in FSCN family, including FSCN1, FSCN2 and FSCN3, which are encoded by *FSCN1, FSCN2* and *FSCN3* gene, respectively 10. The expression of FSCN1 was low or absent from adult epithelia, but often highly increased in many aggressive carcinomas, such as breast cancer 11, pancreatic cancer 12 and hepatocellular carcinoma 13. FSCN1 has been proved to play an important role in promoting metastasis of tumors 14-17. For example, upregulated FSCN1 expression in oral squamous cell carcinoma (OSCC) derived cells resulted in a significant increase in cell migration and invasion. FSCN1 overexpression was significantly correlated with advanced tumor stage and lymph node metastasis in OSCC 18. Moreover, high FSCN1 expression was strongly associated with poor clinical outcomes and could be used as a prognostic and predictive biomarker in different cancer types, including nonsmall cell lung cancer 19, urinary bladder urothelial carcinoma 20 and breast cancer 21, 22. FSCN1 has been extensively studied in recent years, whereas very little is known about FSCN2 and FSCN3. It has been reported that *FSCN2* and *FSCN3* may function in progressive hearing loss 23 and terminal elongation of the spermatid head 24, respectively.

However, the role of FSCN family in MM is still unclear. In this study, we enrolled 1201 patients from four independent GEO datasets and investigated the potential prognostic role of *FSCN* family in MM by exploring the relationships between *FSCN1-3* expression profiles and the clinical outcomes of MM patients.

## Materials and Methods

### Patients

All clinical, cytogenetic and molecular information, as well as gene microarray expression data used in this study were collected from Gene Expression Omnibus (GEO) datasets (http://www.ncbi.nlm.nih.gov/geo). We divided all the samples into two cohorts. The first cohort was used for microarray expression analysis, including GSE39754 (6 normal donors, 170 MM) and GSE2113 (7 MGUS, 39 MM, 6 PCL). The gene expression data was analyzed by Affymetrix Human Genome U133 Plus 2.0 Array. The second cohort was mainly applied for survival analysis. This cohort consisted of two independent microarray datasets of MM patients, GSE24080 and GSE4581. The gene expression profiling of 559 newly diagnosed MM patients in GSE24080 and 414 untreated MM patients in GSE4581 were also evaluated by the Affymetrix Human Genome U133 Plus 2.0 Array.

Clinical endpoints of this study were event-free survival (EFS) and overall survival (OS). EFS was defined as the length of time from diagnosis to the first event, including progression, relapse, death, etc. OS was defined as the length of time from diagnosis to death or the end of the follow-up for any reason.

All experiment design, quality control, and data normalization were in line with the standard Affymetrix protocols. The research was conducted in accordance with the International Conference and the Declaration of Helsinki.

### Statistical analysis

The clinical and molecular characteristics of patients were described using median and/or range. Comparison of numerical data and categorical data were based on the Wilcoxon rank sum test, Kruskal-Wallis test and Fisher exact test, respectively. The Kaplan-Meier methods and log-rank test were applied for survival analysis. Co-expression analysis was conducted by calculating Pearson's correlation coefficient. Univariate and multivariate Cox proportional hazard models were constructed for EFS and OS, using a limited backward elimination procedure. The confidence interval is 95%. All statistical analysis was performed by R software 3.5.0.

## Results

### The expression levels of *FSCN* family in normal donors and myeloma patients in different stages

**Figure 1:**
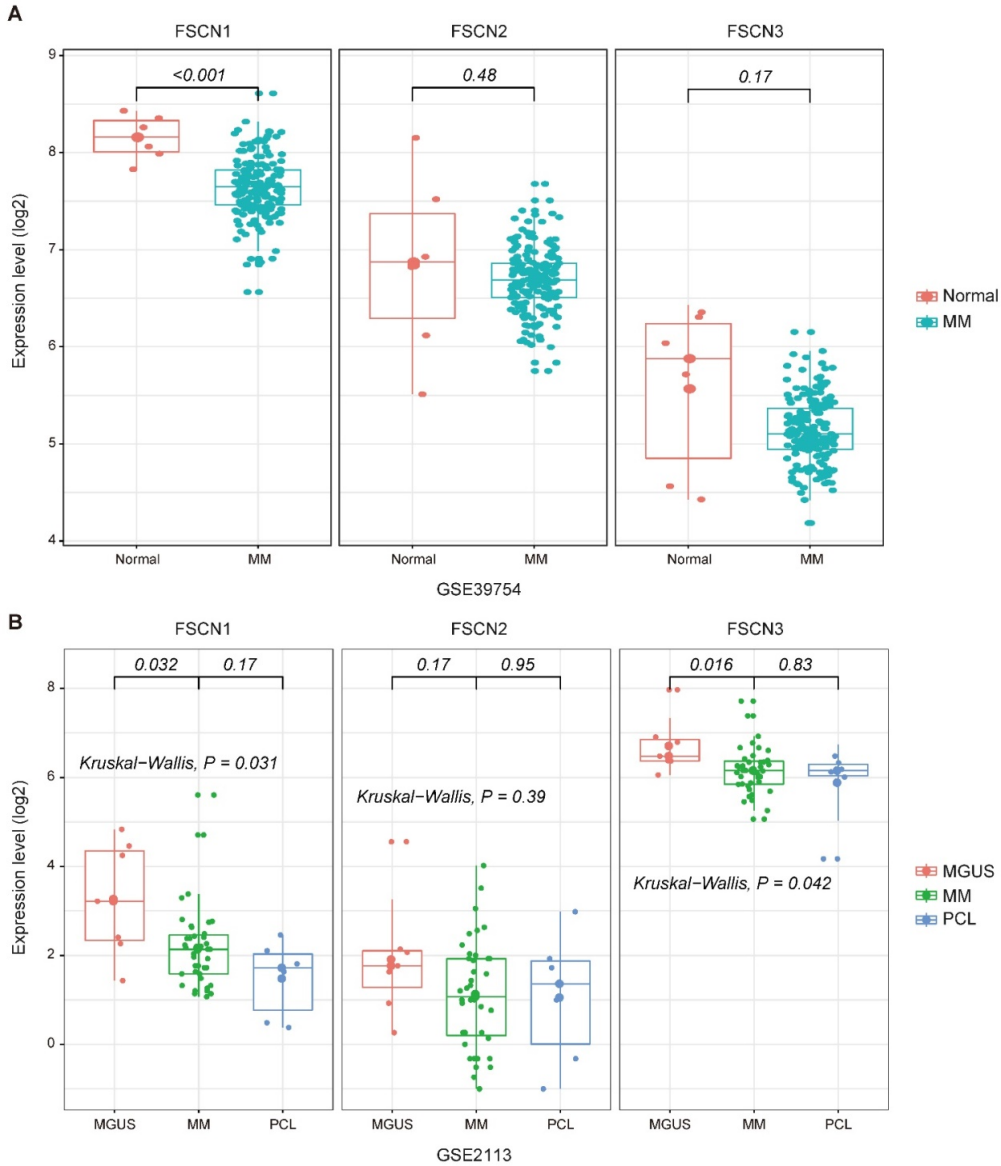
** The expression levels of *FSCN1-3* in normal donors and myeloma patients in different stages.** *X*-axis represents the sample type; Y-axis represents the *FSCN1-3* expression levels (log2). **A** MM patients (n=170) compared with normal donors (n=6) in GSE39754. **B** Comparison of *FSCN1-3* expression levels in three different stages of myeloma patients: MGUS (n=7), MM (n=39), PCL (n=6) in GSE2113.

To investigate the association between expression levels of* FSCN1-3* and MM, we analyzed expression levels of *FSCN1-3* in normal donors and MM patients from GSE39754 dataset. The *FSCN1* expression in MM patients demonstrated a remarkable decrease compared to normal donors (*p* < 0.001, Fig 1A). However, there was no significant difference in the expression of *FSCN2* and* FSCN3* between normal donors and MM patients (Fig 1A).

To explore the relationship between *FSCN1-3* expression levels and the progression of myeloma, we also analyzed *FSCN1-3* expression levels of patients from GSE2113 in three different myeloma stages, including MGUS, MM and PCL. The *FSCN1* and *FSCN3* expression were also down-regulated in MM compared with MGUS (*p* = 0.032, 0.016, Fig 1B), no statistically significance was found between MM and PCL. There was no significant difference in *FSCN2* expression among different myeloma stages (Fig 1B).

### Comparison of EFS and OS between different expression levels of *FSCN* family

**Figure 2:**
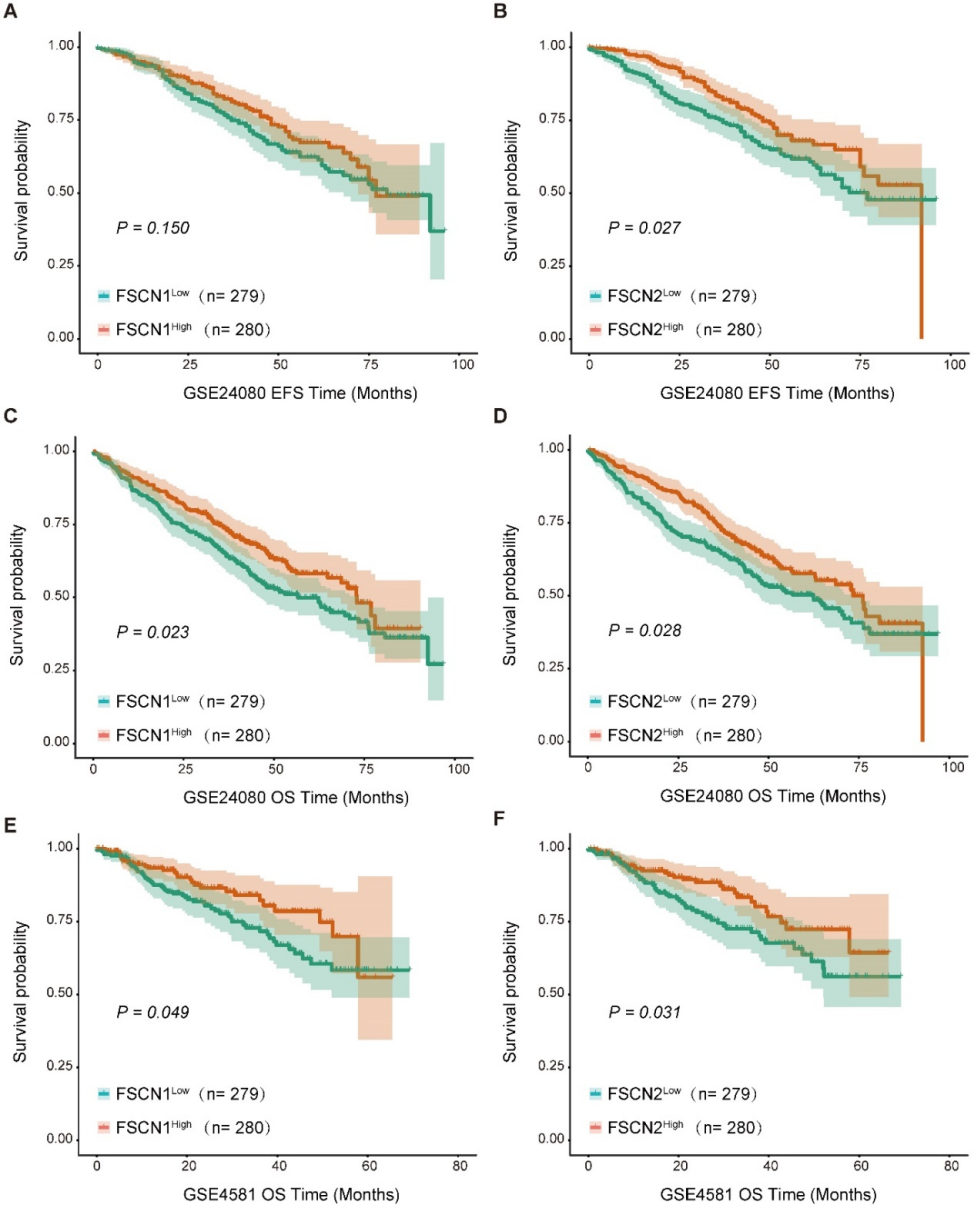
** Survival analysis between different expression levels of *FSCN1* and* FSCN2*. A** No significant difference was observed in EFS between *FSCN1*^high^ group and *FSCN1*^low^ group in GSE24080. **B** *FSCN2*^high^ group had longer EFS than *FSCN2*^low^ group in GSE24080. **C** *FSCN1*^high^ group had longer OS than *FSCN1*^low^ group in GSE24080. **D** *FSCN2*^high^ group had longer OS than *FSCN2*^low^ in GSE24080. **E** *FSCN1*^high^ group had longer OS than *FSCN1*^low^ group in GSE4581. **F** *FSCN2*^high^ group had longer OS than *FSCN2*^low^ in GSE4581.

Using the GSE24080 dataset (559 MM patients), we analyzed the impact of *FSCN1-3* expression on clinical outcomes in MM. Based on the median expression level of each *FSCN* member, we divided all the patients into low and high *FSCN* expression groups. The comparison of EFS and OS between different *FSCN* expression groups were shown in Table 1. High expression of *FSCN1* was significantly associated with longer OS (*p* = 0.023, Fig 2C), and it had no obvious impact on EFS (*p* = 0.150, Fig 2A). EFS and OS in MM patients with high *FSCN2* expression were longer than those with low *FSCN2* expression (*p* = 0.027, 0.028, Fig 2B, Fig 2D). The expression level of *FSCN3* had no effect on EFS and OS of patients in two groups (Table 1). The impacts of elevated levels of *FSCN1* and* FSCN2* on longer OS were also validated in another independent dataset GSE4581 (*p* = 0.049, 0.031, Fig 2E, 2F)*.*

### Gene co-expression analysis for *FSCN* family in MM

**Figure 3:**
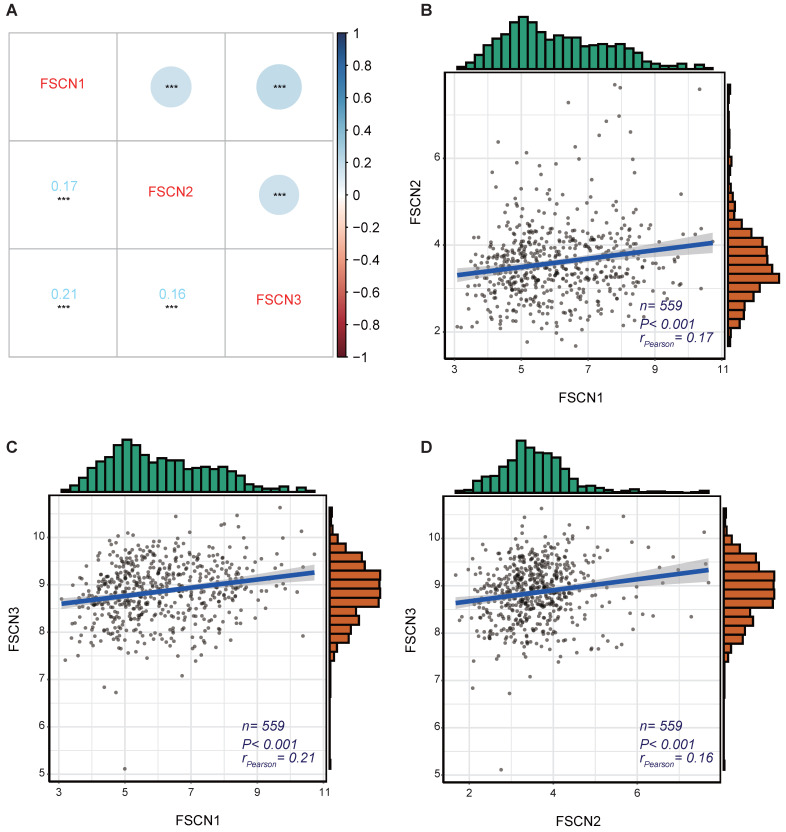
** The gene co-expression analysis for* FSCN* family members in GSE24080 dataset. A** Co-expression heat map of *FSCN* genes. **B** Co-expression relationship between *FSCN1* and *FSCN2.* **C** Co-expression relationship between *FSCN1* and *FSCN3*. **D** Co-expression relationship between *FSCN2* and *FSCN3.*

To identify the expression correlations between *FSCN* family members, we performed a gene co-expression analysis of 559 MM patients in GSE24080 dataset. As shown in Fig 3, the expressions of *FSCN1-3* were not significantly associated with each other (all *r_Pearson_* < 0.5, Fig 3).

### Comparison of clinical and molecular characteristics in different *FSCN1* and *FSCN2* expression

Comparison of the clinical and molecular characteristics of the 559 MM patients in GSE24080 based on different *FSCN1* and *FSCN2* expression levels were summarized in Table 2. Compared to *FSCN1*^low^ group, *FSCN1*^high^ group had decreased beta-2 microglobulin (B2M) level (*p* = 0.015), elevated hemoglobin (HGB) level (*p* < 0.001), less aspirate plasma cells (ASPC) (*p* < 0.001), less bone marrow biopsy plasma cells (BMPC) (*p* < 0.001) and less frequent cytogenetic abnormality (*p* < 0.001). As the same as *FSCN1*, *FSCN2*^high^ group had decreased B2M level (*p* = 0.007) and less frequent cytogenetic abnormality (*p* = 0.001) compared with *FSCN2*^low^ group. In addition, *FSCN2*^high^ group was related to more older patients (*p* = 0.020), more male patients (*p* = 0.028), decreased lactate dehydrogenase (LDH) level (*p* < 0.001) and higher *FGFR3* expression (*p* = 0.009).

### Univariate and multivariate analysis of possible prognostic factors in MM

To further confirm the potential prognostic value of *FSCN* family in MM, age (≥ 60 vs. < 60 years), gender, albumin (ALB), B2M, HGB, LDH, expression levels of *FSCN1-3* and other common genetic mutations (*CCND1, FGFR3, LIG4,* and* TP53*) were included in univariate and multivariate cox regression analysis.

As shown in Table 3, univariate analysis demonstrated that ALB (*p* = 0.033, < 0.001), B2M (both *p* < 0.001), HGB (*p* < 0.001, = 0.002), LDH (both* p* < 0.001),* FSCN2* expression (*p* = 0.028, = 0.018) were significantly correlated with both EFS and OS of 559 MM patients in GSE 24080. Additionally,* CCND1* expression (*p* = 0.006) was significantly associated with EFS. Age (*p* = 0.028) and *FSCN1* expression (*p* = 0.003) were closely related to OS in univariate analysis. While in multivariate analysis, LDH was an independent risk factor for both EFS and OS (both *p* < 0.001). For EFS, HGB (*p* = 0.023) and *CCND1* (*p* = 0.011) were independent favorable factors. As for OS, ALB (*p* < 0.001), *FSCN1* (*p* = 0.002) and *FSCN*2 (*p* = 0.013) were independent favorable factors, while age (*p* = 0.029), B2M (*p* = 0.010) were independent risk factors.

### The combined prognostic significance of *FSCN1* and *FSCN2* in MM

**Fig 4:**
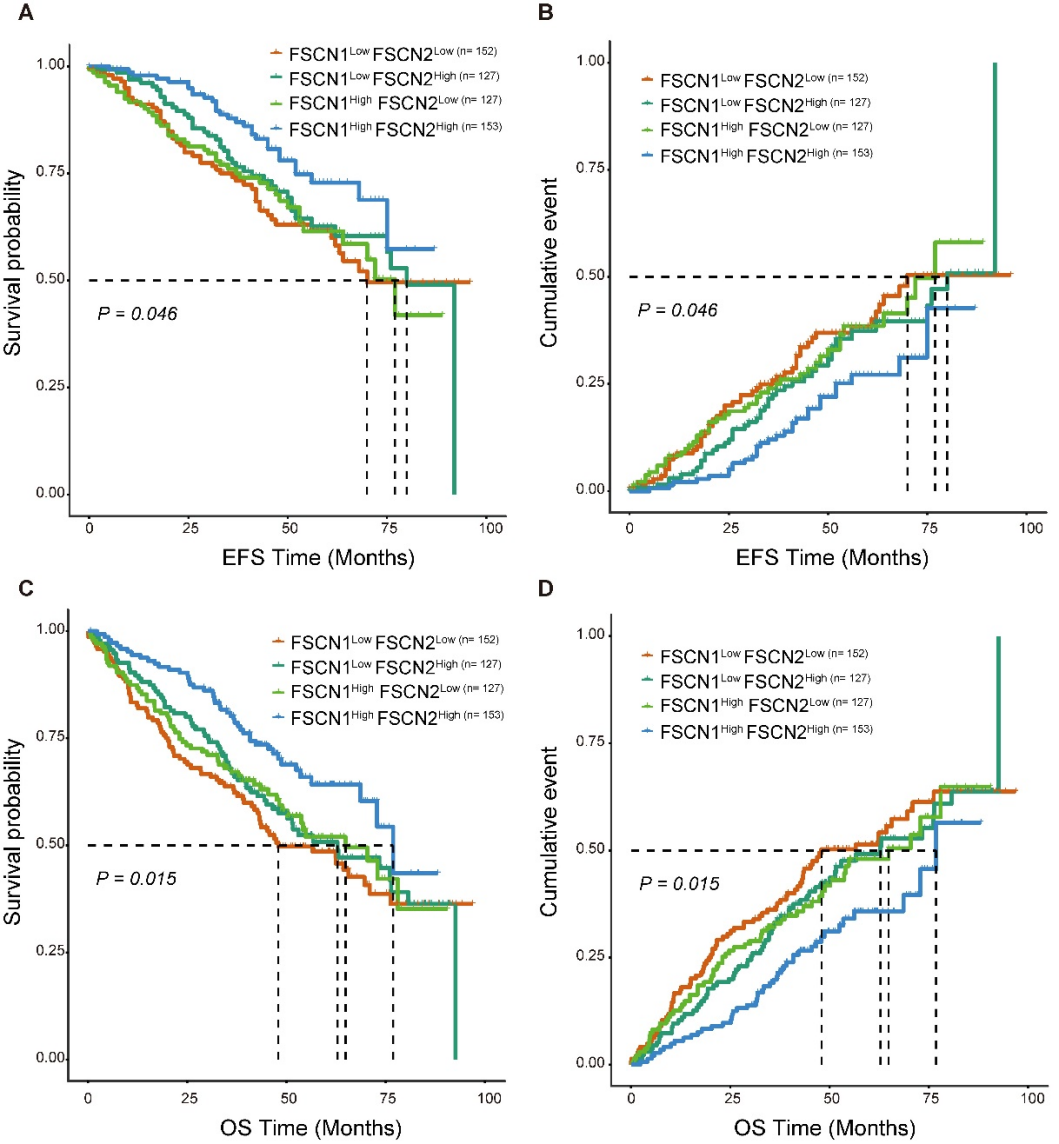
**Survival analysis of combination of different expression levels of *FSCN1* and* FSCN*2 in GSE24080 dataset. A, B** *FSCN*1^high^* FSCN*2^high^ group had longer EFS than other groups.** C, D** *FSCN*1^high^* FSCN*2^high^ group had longer OS than other groups.

As *FSCN1* and *FSCN2* were proved to be independent prognostic factors in MM, we further explored their combined prognostic significance in 559 patients from GSE24080. As shown in Fig 4, *FSCN1*^high^* FSCN2*^high^ group had significant longer EFS and OS compared to the other three groups (*p* = 0.046, 0.015).

## Discussion

In this study, we found that the expression levels of *FSCN1* and *FSCN*3 were significantly decreased in MM. Enhanced expressions of *FSCN1* and* FSCN2* closely related to longer OS and could serve as independent favorable prognostic factors for OS in MM. Combining high expression of *FSCN1* and* FSCN2* could not only effectively predict longer OS but also longer EFS.

*FSCN1* was usually up-regulated in many malignant tumors and could be considered as an oncogene by promoting migration and invasion of tumors cells 25. Increasing evidences suggested that elevated level of FSCN1 was significantly correlated with increased metastatic potential and more aggressive phenotypes in a variety of tumors 26-29, and inhibiting FSCN1 could block the migration and metastasis of tumor cells 30. For instance, the increased expression of FSCN1 in HR-negative breast cancers might contribute to their more aggressive behavior 11, down-regulation of *FSCN1* by si-RNA dramatically reduced the migratory abilities of breast cancer cells 31. Forced expression of FSCN1 in cultured colorectal cancer cells promoted their migratory and invasive capabilities *in vitro* and enabled cells had higher abilities to form metastases *in vivo*, whereas specific inhibition of FSCN1 expression reduced colorectal cancer cells invasion 32. The anti-migration and anti-invasion effect by knocking-down expression of FSCN1 could also be found in ovarian cancer 33, non-small cell lung cancer 34 and glioblastoma 35. Thus, inhibition of *FSCN1* expression may be essential for anti-metastatic therapy. Additionally, the increased expression of* FSCN1* has been proved to be an adverse biomarker predicting poor outcomes in many types of malignancies 36, 37. Surprisingly, in contrast to most of malignancies, *FSCN1* was found to be down-regulated in two independent GSE datasets in MM (GSE39754 and GSE2113, Fig 1), and high expression of *FSCN1* was closely related to longer OS in MM, which was confirmed in 973 patients from GSE24080 and GSE4581 (Table 1, Fig 2). This unique inverse correlation between the expression of *FSCN1* and the prognosis of MM patients is unexpected and needs further investigation. In addition, compared with *FSCN1*^low^ group, patients with high expression of* FSCN1* had decreased levels of unfavorable prognostic factors (B2M, ASPC, BMPC and cytogenetic abnormality) and increased level of favorable one (HGB) (Table 2), which might partially contribute to longer OS in *FSCN1*^high^ group. In multivariate analysis, we proved that *FSCN1* can be an independent favorable prognosis factor for OS (*p* = 0.002, Table 3) Further investigation is required to evaluate using *FSCN1* as a therapeutic target in MM.

Previous studies on *FSCN2* have focused on the role of maintaining ear and eye functions 23, 38, 39. Very little was found in the literature on the relationship between *FSCN2* and tumors. In this study, we demonstrated that high expression of *FSCN2* was significantly associated with favorable EFS and OS in MM (Table 1, Fig 2). We also found that B2M, LDH, cytogenetic abnormality, which were related to poor clinical outcomes in MM, showed a significant decrease in *FSCN2*^high^ group compared to *FSCN2*^low^ group (Table 2). To further confirm whether *FSCN2* could predict prognosis independently, multivariate analysis was conducted and high expression of *FSCN2* was proved to be an independent positive prognosis indicator for OS in MM (*p* = 0.013, Table 3). Further efforts are required to explore how *FSCN2* affects the patient survival.

As *FSCN1* and *FSCN2* were both positively related to OS, we further investigated the prognostic role of the combination of *FSCN1* and *FSCN2*. *FSCN1* and *FSCN2* did not show a coordinated expression pattern in our study (*r_Pearson_* < 0.5, Fig 3). This was in line with previous studies that *FSCN1* was expressed in neural and mesenchymal tissues and *FSCN*2 was predominantly expressed in retinal photoreceptor cells, respectively 28. As to the prognosis, combination of high expression of *FSCN1* and* FSCN2* could not only effectively predict longer OS but also longer EFS (all *p* < 0.05, Fig 4).

In multivariate analysis, consistently with previous studies, we found that LDH was an independent risk factor for both EFS and OS, B2M was an independent risk factor for OS, and HGB was an independent favorable factor for EFS (Table 3). Cyclin D1 (CCND1) is a critical modulator in cell cycle. The prognostic role of* CCND1* in MM is still controversial. *CCND*1 was reported to be associated with unfavorable prognosis in MM 40, 41, whereas it was identified as a favorable prognostic indicator in another study 42. In our study, we showed that *CCND1* was an independent favorable factor for EFS in MM (Table 3).

In conclusion, our research demonstrated that increased expression levels of *FSCN1* and *FSCN2* were strongly associated with longer OS and they were independent favorable prognostic factors for OS in MM. In addition, the combination of *FSCN1* and *FSCN2* expression was an effective prognosis predictor for both EFS and OS in MM. However, the related molecular mechanism of *FSCN* family in MM remains unclear and needs to be further investigated.

## Figures and Tables

**Table 1:** Comparison of EFS and OS between the high and low expression levels of* FSCN* family in GSE24080.

	EFS		OS	
	*χ2*	*p-*value	*χ2*	*p-*value
FSCN1(High vs. Low)	3.170	0.075	12.976	<0.001
FSCN2 (High vs. Low)	3.861	0.049	5.379	0.020
FSCN3 (High vs. Low)	1.505	0.220	3.131	0.077

Abbreviations: EFS: event-free survival; OS: overa ll survival.

**Table 2:** Patients' characteristics of 559 multiple myeloma patients in GSE24080.

	*FSCN1*			*FSCN2*		
	Low (n = 279)	High (n = 280)	*p-*value	Low (n = 279)	High (n = 280)	*p-*value
Age, mean (range)	56.92 (29.7-76.5)	57.44 (24.83-75)	0.200	56.31 (24.83-76.5)	58.05 (30.5-75)	0.020
Gender, no (%)						
female	116 (41.58)	106 (37.86)	0.417	124 (44.44)	98 (35)	0.028
male	163 (58.42)	174 (62.14)		155 (55.56)	182 (65)	
Race, no (%)						
other	31 (11.11)	31 (11.07)	1.000	37 (13.26)	25 (8.93)	0.135
white	248 (88.89)	249 (88.93)		242 (86.74)	255 (91.07)	
ISS, no (%)						
I	171 (61.07)	147 (52.69)	0.091	169 (60.36)	149 (53.41)	0.078
II	59 (21.07)	63 (22.58)		62 (22.14)	60 (21.51)	
Ⅲ	50 (17.86)	69 (24.73)		49 (17.5)	70 (25.09)	
B2M (mean(sd))	5.29 (6.295)	4.19 (4.171)	0.015	5.351 (6.209)	4.129 (4.281)	0.007
CRP (mean(sd))	12.328 (26.743)	10.934 (18.34)	0.473	11.698 (17.113)	11.563 (27.534)	0.944
CREAT (mean(sd))	1.36 (1.322)	1.286 (1.216)	0.494	1.426 (1.438)	1.219 (1.068)	0.054
LDH (mean(sd))	171.065 (71.332)	172.886 (60.189)	0.744	181.828 (73.813)	162.161 (55.424)	<0.001
ALB (mean(sd))	4.042 (0.559)	4.056 (0.605)	0.780	4.056 (0.585)	4.042 (0.58)	0.774
HGB (mean(sd))	10.961 (1.794)	11.545 (1.785)	<0.001	11.104 (1.752)	11.401 (1.86)	0.052
ASPC (mean(sd))	47.842 (23.14)	37.568 (23.249)	<0.001	44.143 (24.639)	41.247 (22.702)	0.149
BMPC (mean(sd))	52.326 (25.13)	40.515 (25.38)	<0.001	48.5 (26.52)	44.298 (25.137)	0.055
MRI (mean(sd))	9.962 (12.728)	12.111 (15.194)	0.070	12.062(14.582)	10.049 (13.443)	0.090
Cytogenetic abnormality (%)						
No	151 (54.12)	201 (71.79)	<0.001	157 (56.27)	195 (69.64)	0.001
Yes	128 (45.88)	79 (28.21)		122 (43.73)	85 (30.36)	
ISOTYPE, no (%)						
FLC	52 (18.57)	32 (11.47)	0.112	35(12.5)	49 (17.56)	0.694
IgA	57 (20.36)	76 (27.24)		67(23.93)	66 (23.66)	
IgD	2 (0.71)	1 (0.36)		1(0.36)	2 (0.72)	
IgG	156 (55.71)	157 (56.27)		165(58.93)	148 (53.05)	
Nonsecretory	3 (1.07)	3 (1.08)		2(0.71)	4 (1.43)	
NSE	2 (0.71)	0 (0)		1(0.36)	1 (0.36)	
High CCND1, no (%)	138 (49.46)	142 (50.71)	0.565	135 (48.39)	145 (51.79)	0.679
High LIG4, no (%)	139 (49.82)	141 (50.36)	0.746	140 (50.18)	140 (50)	0.308
High TP53, no (%)	150 (53.76)	130 (46.43)	0.378	143 (51.25)	137 (48.93)	0.620
High CDK4, no (%)	147 (52.69)	133 (47.5)	0.312	152 (54.48)	128 (45.71)	0.651
High FGFR3, no (%)	140 (50.18)	140 (50)	0.213	113 (40.5)	167 (59.64)	0.009
High CDK5, no (%)	138 (49.46)	142 (50.71)	0.943	143 (51.25)	137 (48.93)	0.721
High HK2, no (%)	152 (54.48)	128 (45.71)	0.478	145 (51.97)	135 (48.21)	0.142

Abbreviations: ALB: abumin (35 g/l); ASPC: Aspirate plasma cells (%); BMPC: Bone marrow biopsy plasma cells (%); B2M: beta-2 microglobulin (mg/l); CREAT: creatinine (mg/dl); CRP: C-reactive protein (mg/l); HGB: hemoglobin (g/dl); ISS: International Staging System; LDH: lactate dehydrogenase (U/l); MRI: number of magnetic resonance imaging (MRI)-defined focal lesions (skull, spine, pelvis); no: number of patients.

**Table 3:** Univariate and multivariate cox regression analysis of EFS and OS in 559 multiple myeloma patients.

	Univariate cox regression				Multivariate cox regression			
	EFS		OS		EFS		OS	
	HR (95%CI)	*p-*value	HR (95%CI)	*p-*value	HR (95%CI)	*p-*value	HR (95%CI)	*p-*value
Age (≥60 *vs.* <60)	0.97 (0.71-1.32)	0.839	1.40 (1.04-1.89)	0.028	0.89 (0.65-1.23)	0.492	1.41 (1.04-1.92)	0.029
Gender	1.05 (0.77-1.43)	0.750	0.97 (0.72-1.32)	0.850	1.34 (0.97-1.86)	0.075	1.24 (0.90-1.71)	0.185
ALB	0.72 (0.53-0.97)	0.033	0.48 (0.35-0.64)	< 0.001	0.76 (0.56-1.04)	0.088	0.51 (0.37-0.70)	< 0.001
B2M	1.72 (1.27-2.33)	< 0.001	2.21 (1.64-3.00)	< 0.001	1.25 (0.88-1.78)	0.211	1.58 (1.12-2.22)	0.010
HGB	0.54 (0.39-0.74)	< 0.001	0.62 (0.45-0.84)	0.002	0.66 (0.46-0.95)	0.023	1.00 (0.70-1.41)	0.980
LDH	2.58 (1.65-4.05)	< 0.001	3.68 (2.53-5.37)	< 0.001	2.31 (1.45-3.68)	< 0.001	3.18 (2.13-4.73)	< 0.001
FSCN1 (High *vs.* Low)	0.80 (0.59-1.09)	0.151	0.63 (0.46-0.85)	0.003	0.83 (0.60-1.15)	0.265	0.60 (0.43-0.82)	0.002
FSCN2 (High *vs.* Low)	0.71 (0.52-0.96)	0.028	0.69 (0.51-0.94)	0.018	0.73 (0.53-1.00)	0.051	0.66 (0.48-0.92)	0.013
FSCN3 (High *vs.* Low)	0.84 (0.62-1.14)	0.254	0.77 (0.57-1.04)	0.093	0.95 (0.68-1.33)	0.768	0.92 (0.66-1.27)	0.607
CCND1(High *vs.* Low)	0.65 (0.48-0.88)	0.006	0.74 (0.55-1.00)	0.053	0.66 (0.48-0.91)	0.011	0.87 (0.64-1.20)	0.400
FGFR3 (High *vs.* Low)	0.90 (0.67-1.22)	0.508	0.80 (0.59-1.08)	0.150	0.98 (0.70-1.36)	0.902	0.93 (0.67-1.28)	0.642
LIG4 (High *vs.* Low)	0.84 (0.62-1.14)	0.268	0.84 (0.63-1.14)	0.269	0.87 (0.64-1.18)	0.364	0.93 (0.68-1.26)	0.636
TP53 (High *vs.* Low)	1.06 (0.78-1.44)	0.700	0.85 (0.63-1.15)	0.284	1.14 (0.83-1.56)	0.415	0.88 (0.65-1.20)	0.418

Abbreviations: ALB: albumin 35 g/l; B2M: beta-2 microglobulin mg/l; CR: complete remission; CI: confidence interval; EFS: event-free survival; HGB: hemoglobin g/dl; HR: hazard ratio; LDH: lactate dehydrogenase U/l; OS: overall survival.
